# Vitamin D Receptor TaqI Gene Variant in Exon 9 and Polycystic Ovary Syndrome Risk

**Published:** 2013-07-31

**Authors:** Morteza Bagheri, Isa Abdi Rad, Nima Hosseini Jazani, Fariba Nanbakhsh

**Affiliations:** 1 Food and Beverages Safety Research Center, Urmia University of Medical Sciences, Urmia, Iran; 2 Department of Genetics, Urmia University of Medical Sciences, Urmia, Iran; 3 Cellular and Molecular Research Center, Urmia University of Medical Sciences, Urmia, Iran; 4 Reproductive Health Research Center, Department of Obstetrics and Gynecology, Urmia University of Medical Sciences, Urmia, Iran

**Keywords:** Vitamin D Receptor, Polycystic Ovary Syndrome, Genetic Variation

## Abstract

**Background:**

Polycystic ovary syndrome (PCOS) is known as a metabolic disorder. The results of recent studies implied that vitamin D receptor (VDR) genetic variants may impact PCOS and insulin resistance in women with PCOS. The aim of the present study was to determine the VDR TaqI gene variant in exon 9 (T/C) (rs731236) in normal controls and patients with PCOS for the first time in Iranian Azeri women.

**Materials and Methods:**

In this case control study between April 2011 and June 2012, a total of 76 women aged 18-40 years (38 patients with PCOS and 38 healthy women as normal controls) participated. Genotypes of VDR TaqI in exon 9 (T/C) (rs731236) were determined using the PCR-RFLP method.

**Results:**

The frequencies of VDR TaqI T anc C alleles were 0.605 and 0.395 in cases and 0.697 and 0.303 in controls. Also, the genotypic frequencies of VDR TaqI were 16) (42.11), 14(36.84), and 8(21.05) in cases, and 17(44.74), 19(50), and 2(5.26) in controls for TT, TC and CC genotypes respectively. There was no difference in genotype and allele frequencies between PCOS and controls (p value>0.05) with the exception of the CC genotype (p value=0.04).

**Conclusion:**

This report, a first of its own kind in Iranian Azeri patients, suggests that the CC genotype of VDR TaqI in exon 9 (rs731236) is associated with PCOS.

## Introduction

Polycystic ovary syndrome (PCOS) is the most common multifactorial disorder of the endocrine system (1). Etiological studies have shown that several complicating factors play critical roles in the pathogenesis of PCOS (2). Numerous studies have demonstrated that vitamin D deficiency leads to insulin resistance (IR) as well as type 2 diabetes melitus. IR has been involved in the pathogenesis of PCOS (3-5). It has been suggested that supplementation of vitamin D has a determining role in treatment of PCOS (6). Vitamin D as a steroid hormone is the main regulator of calcium homeostasis by converting to the active hormone 1, 25-dihydroxycholecalciferol in the liver and kidneys (7-9). Vitamin D functions are mediated via the vitamin D receptor (VDR).

The results of recent studies implied that VDR genetic variants may impact PCOS and IR in women with PCOS (8, 9). Vitamin D and calcium repletion predict reproductive success following fertilization (10, 11). Regulation of the egg activation, oocyte maturation, follicular development and mammalian embryo development is Ca^2+^ dependent (12). The VDR is defined as the nuclear steroid hormone receptor (13) resulting in gene expression regulation through binding to specific response elements within the promoter of some genes (13, 14). Cellular ligandactivated transcription factors are encoded by the VDR gene (15). These transcription factors have different functions including calcium homeostasis. The mechanism of gene expression regulation by VDR is not well characterized. It has been demonstrated that VDR produces a specific protein which interacts with the basal transcription factor TFIIB (15). Also, VDR regulates gene transcription with other different mechanisms including interaction with co-activator or co-repressor molecules. VDR may influence the acetylation of histones as well as chromatin remodeling (16-21).

VDR gene contains 14 exons and is mapped on chromosome 12cen-q12. Several allelic variations have been reported in the VDR gene such as the following restriction fragment length polymorphisms: FokI in exon 2 (C/T) (rs10735810), BsmI in intron 8 (G/A) (rs1544410), ApaI in intron 8 (C/A) (rs7975232), Tru9I in intron 8 (G/A) (rs757343) and TaqI in exon 9 (T/C) (rs731236). TaqI based restriction fragment length polymorphism is located at the 3' end of the VDR gene. The function of the TaqI-specific hypervariable polymorphism is unclear (22). VDR gene variants have been associated to breast cancer risk (23), prostate cancer progression (24), colorectal cancer (25), diabetes (26-28), primary hyperparathyroidism (29), coronary artery disease (30) and PCOS (5, 31-34). Grulet et al. (33) indicated that there is an association between insulin resistance and hyperandrogenism as well as luteinizing hormone (LH) and insulin sensitivity in PCOS (33, 34).

The findings of Ranjzad et al. (5) demonstrated that there is a significant association between VDR TaqI CC genotype and serum concentrations of LH in women with PCOS. Whereas there is a association between VDR TaqI CC genotype with serum level of LH (5) and LH with insulin sensitivity in PCOS (33, 34), the aim of present study was to determine the VDR TaqI gene variant in exon 9 (T/C) (rs731236) in normal controls and patients with PCOS for the first time in Iranian Azeri women.

## Materials and Methods

This matched case-control study was approved by the Ethical Committee of Urmia University of Medical Sciences and was performed in Urmia University of Medical Sciences (Urmia, Iran).

Between April 2011 and June 2012, a total of 76 women aged 18-40 years (38 patients with PCOS and 38 healthy women as normal controls) participated in this study. Women with PCOS and controls were unrelated and matched for gender, age, ethnicity, height, weight, and geographical region. Women with PCOS were clinically examined in assisted reproductive technology (ART) Reproductive Center and Infertility Clinic by ART and infertility specialists. Familial and medical history, physical evaluations, and clinical tests were carried out by the same physicians for all participants.

All diagnosis was based on the finding of three or more of the criteria proposed by the Rotterdam criteria (35) and on the basis of the National Institute of Child Health and Human Development (NICHD) criteria (36). PCOS was confirmed by the presence of menstrual disorder including oligomenorrhea (six or fewer menses per year), amenorrea (no mense in the last six months), hyper-androgenism (clinical-biochemical signs) such as hirsutism (Ferriman-Gallwey score ≥6), acne, or alopecia as well as elevated androgen levels (testosterone normal range <0.77 ng/ ml and free testosterone normal range <3.18 pg/ml).

Women with PCOS and controls without taking any medications known to influence the endocrinal system, carbohydrate, lipid and calcium metabolism for at least three months before entering the investigation were studied. Women, both cases and controls, with a history of any known cause of oligomenorrhea, amenorrea, hyper-androgenism including non-classic congenital adrenal hyperplasia, hyper-prolactinaemia and other confounding factors such as diabetes, Cushing’s syndrome, androgen-producing tumours, pregnant or breast feeding females, thyroid dysfunction, cases with vitamin D supplementation as well as individuals taking medications that affect calcium homeostasis such as corcicosteroids, anticonvulsants were excluded from the study (5).

Before blood sampling, informed written consent was taken from all participants. A 3-5 mL aliquot of peripheral blood was collected with ethylenediaminetetraacetic acid (EDTA)-containing tubes for isolation of genomic DNA by standard salting out method (37).

Genotypes of VDR TaqI in exon 9 (T/C) (rs731236) were determined using the PCR-RFLP method in controls and patients with PCOS (5). The optimized primer pair sequences were 5′-CAG AGC ATG GAC AGG GAG CAA G-3′ and 5′- GCA ACT CCT CAT GGC TGA GGT CTC A-3′ (5). PCR procedure was carried out via primary denaturation at 94˚C for 5 minutes, and then followed by 35 cycles of 45 sec at 93˚C, 30 seconds at 66˚C and 45 seconds at 72˚C. Final extension reaction was performed for 10 minutes at 72˚C (5). Each PCR reaction was carried out in a 20 μl solution containing 100 ng of genomic DNA, 1x reaction buffer, 10 pmol of each primer, 200 μmol of each dNTPs, 0.5 unit of Taq DNA polymerase, and 1.5 mmol MgCl2. PCR amplicons of VDR/TaqI were 740 bp in length and after digestion with TaqI enzyme at 65˚C for 2 hours produced fragments of 495 + 245 bp for "T" and 290 + 245 + 205 bp for "C" alleles respectively. In the heterozygote state, VDR TaqI in exon 9 (T/C) (rs731236) produced four fragments (205, 245, 290 and 495 bp), in homozygote VDR TaqI in exon 9 (C/C) (rs731236) three fragments (205, 245, and 290 bp) and in homozygote VDR TaqI in exon 9 (T/T) (rs731236) two fragments (245 and 495 bp) could be detected.

Electrophoresis of PCR products and digested fragments was carried out on 2.5% agarose gel, and presence or absence of fragments were visualized by the UV transilluminator. The frequencies of VDR TaqI gene variant were computed by direct counting regarding T and C alleles and TT, TC, and CC genotypes in normal controls and patients with PCOS.

The frequencies of alleles and genotypes in the tested patient group were compared with the control group by using a Χ^2^ test. The data were analyzed regarding their fitness to Hardy-Weinberg equilibrium (HWE). A minimum sample size of 36 patients in cases had a statistical power of about 70% (two-tailed, α=0.10). The SPSS ver. 16.0 software and Microsoft Office Excel 2007 were used to calculate the Χ^2^ value, the odds ratio (OR), and 95% confidence interval (CI) as well as analysis of independent t test for detection of differences between cases and controls regarding clinical characteristics. P value less than 0.05 was considered statistically significant.

## Results

The clinical characteristics of women with PCOS are summarized in table 1. The studied group consisted of 38 Iranian Azeri PCOS patients (age range: 18-36 years; mean ± SD age, 26.03 ± 4.98) and 38 healthy women (age range: 20-40 years; mean ± SD age, 27.18 ± 4.95) (p>0.05).

**Table 1:** The clinical characteristics of PCOS women


	Cases	Controls

**Number of subjects**	38	38
**Age (Y)**	26.03 ± 4.98	27.18 ± 4.95
**Height (cm)**	160.61 ± 6.69	162.37 ± 6.44
**Weight (kg)**	74.01 ± 12.50	68.80 ± 12.16
**BMI (kg/m^2^)**	28.56 ± 3.65	26.04 ± 3.90


Data presented as means ± SD. BMI, body mass index.

There was no significant difference between women with PCOS and controls regarding clinical characteristics such as height and weight (p>0.05). There was a significant difference between women with PCOS and controls with respect to BMI (kg/ m^2^) (p<0.05). Our cases had a normal report on their blood tests regarding total testostrone level. Percentage of hirsutism and obesity (BMI>27 kg/m^2^) in tested patients were about 100% and 61.53%, respectively. LH and follicle-stimulating hormone (FSH) levels were equal to 7.52 ± 4.02 (IU/l) and 5.36 ± 1.89 (mIU/ml) respectively. The LH/FSH ratio was 1.44 ± 0.62 in our tested PCOS patients.

Allelic and genotypic frequencies of VDR TaqI polymorphism in exon 9 (T/C) (rs731236) are shown in table 2. Statistical analysis showed that cases (Χ^2^=1.99, p=0.369) and controls (Χ^2^=1.29, p= 0.523) were in agreement with HWE. The allele frequencies of VDR TaqI polymorphism were 0.605 and 0.395 in cases, and 0.697 and 0.303 in controls regarding T and C alleles. Also, the genotypic frequencies of VDR TaqI were 16 (42.11), 14 (36.84), and 8 (21.05) in cases, and 17 (44.74), 19 (50), and 2 (5.26) in controls regarding TT, TC, and CC genotypes, respectively.

**Fig 1:**
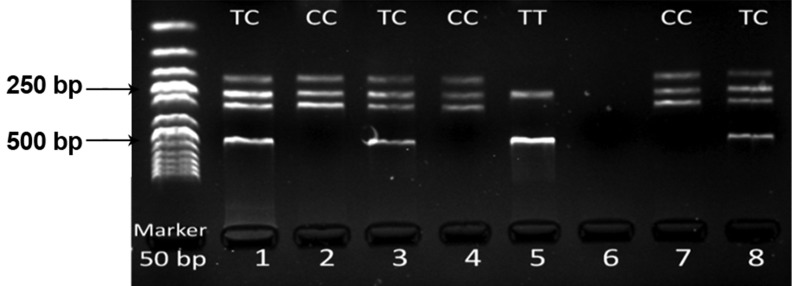
Detection of VDR TaqI genotypes (T/C) via PCR-RFLP in 7 samples.

There was no difference in genotype and allele frequencies between PCOS and controls (Table 1); but the only exception was the CC genotype of VDR TaqI in exon 9 (rs731236) (p value=0.041). VDR TaqI genotypes in exon 9 using electrophoresis are shown in figure 1.

**Table 2:** Allelic and genotypic frequencies of VDR TaqI in exon 9 (T/C) (rs731236) in cases and controls


VDR TaqI T/C	F (%F) cases	F (%F) controls	OR (95% CI)	Χ^2^	P value

**TT**	16 (42.11)	17 (44.74)	0.89 (0.36-2.22)	0.05	0.816
**TC**	14 (36.84)	19 (50)	0.58 (0.23-1.45)	1.33	0.247
**CC**	8 (21.05)	2 (5.26)	4.8 (0.94-24.33)	4.14	0.041
**T**	46 (60.53)	53 (69.74)	0.66 (0.33-1.30)	1.41	0.233
**C**	30 (39.47)	23 (30.26)	1.50 (0.76-2.94)	1.41	0.233


## Discussion

Current understanding of the molecular actions of vitamin D in the reproductivity of women and calcium homeostasis prompted the design of the present study. The goal of this investigation was to study whether the VDR TaqI gene variant in exon 9 (T/C) (rs731236) is related to onset of PCOS for the first time in Iranian Azeri women. PCOS is knwon as a syndrome and affects ovarian function. PCOS most commonly occur during adolescence and characterized by several different features including amenorrhoea, oligomenorrhoea, infertility as well as other metabolic problems in medical findings (38). It has been indicated that some females with syndrome will show polycystic ovary without clinical features of androgen excess. The prevalence of clinical PCOS in high school students in north of Iran was similar to the international estimates of 10-20% in Caucasians according to National Institutes of Health (NIH) criteria (38, 39). A patient with PCOS has an increased risk of obesity, bleeding disorders, hyperandrogenemia, endometrial carcinoma, breast cancer, chronic anovulation, infertility, IR, diabetes, hypertension, primary hyperparathyroidism, dyslipidemia and coronary artery disease (39-41).

IR is in association with reproductive abnormalities in PCOS women. IR is corralated with vitamin D metabolism in PCOS (42). Biological responses and functions of vitamin D are mediated via the VDR within the vitamin D endocrine system in more than 30 target tissues (43-49). In the body, vitamin D regulates calcium homeostasis; an important function in development of the skeletal system as well as in bone mineralization (50). Since vitamin D is the main regulator for calcium and phosphate translocation from the small intestine into the circulation, defects observed in the mutant VDR and calcium absorption lead to decreased level of mineral transport and hypocalcemia (51). Vitamin D and calcium repletion predict reproductive success following fertilization (10, 11). Liang et al. indicated a dynamic role for Ca^2+^ level in oocyte maturation and early embryonic development. Other studies are consistence with Liang et al. and imply that regulation of the egg activation, follicular development and mammalian embryo development are Ca^2+^ dependent (12). The findings of Oh and Barrett-Connor (28) suggest that VDR gene variant may be associated with glucose intolerance independent of defective insulin secretion and with IR. Mahmoudi (32) indicated that VDR gene variant may affect PCOS development as well as IR in women with PCOS. IR and increased levels of LH are usual signs of PCOS (5, 38, 39). High levels of LH not only has an effect on oocyte maturity and human reproduction (52) but also on lower fertility and higher miscarriage prevalence (53). But still there are controversial findings about the action of LH on oocyte, embryo quality, fertility, implantation and miscarriage prevalence (54, 55).

Patients with PCOS show higher levels of LH than constant and lower level of FSH as compared with controls (5, 56, 57). Thus, high levels of LH ranging from 30 to 90 % leads to an elevated ratio of LH/FSH in PCOS (57). Ranjzad et al. (5) demonstrated that VDR TaqI CC genotype has been associated with elevated serum levels of LH in PCOS patients (p=0.011). In this study, the CC genotype of VDR TaqI in exon 9 (rs731236) was significantly higher in cases versus controls (p value=0.04). The findings of the present study was consistent with some reports (5, 28, 31, 33) and inconsistence with others (32) regarding the VDR TaqI gene variant. Some limitations to be considered for the present study include low sample size, poor medical documentations and lack of information regarding vitamin D status in contibuters. Hence, studies in large numbers of PCOS cases are nessecary to validate our results.

## Conclusion

This report suggests that the CC genotype of VDR TaqI in exon 9 (rs731236) is associated with PCOS.
